# Mortality from Alzheimer's Disease and Other Dementias in Ecuador during the Period 2012-2022

**DOI:** 10.2174/0117450179376076250530074402

**Published:** 2025-06-02

**Authors:** Cristobal Espinoza, Maria Salinas, Alicia Morocho, Alex Morales, Byron Verdezoto

**Affiliations:** 1 Catholic University of Cuenca. Research Group, Health, Science, Innovation “ISCI”, Cuenca, Ecuador; 2 Latin American Center for Epidemiological Studies and Social Health, Cuenca, Ecuador

**Keywords:** Alzheimer's disease, Dementias, Mortality, Ecuador, Public health, Mental Health

## Abstract

**Introduction:**

Alzheimer's disease (AD) is a growing concern worldwide in healthcare. In Ecuador, the increasing life expectancy has raised the prevalence of age-related diseases, including dementias. The main objective of this study was to analyze the mortality from AD and other dementias in Ecuador from 2012 to 2022.

**Methodology:**

A retrospective, descriptive time series analysis was conducted on adult subjects with AD and other dementias across various geographic regions of Ecuador during the 2012-2022 period. A sample of 855,122 individuals registered in the databases of the National Institute of Statistics and Censuses (INEC) was analyzed.

**Results:**

Out of the total evaluated subjects, 4,836 deaths were due to AD (0.56%) and 1,317 deaths from other types of dementia (0.15%). For AD, the distribution of deaths by sex showed a predominant trend in women (n=3,008) within the group aged 65 years or older (n=4,749). For other dementias, women were also the main group (n=766), along with those aged 65 years and older (n=1,294). The national mortality rate showed an upward trend during this decade, increasing from 2.2 per 100,000 inhabitants in 2012 to 4.86 per 100,000 inhabitants in 2022.

**Discussion:**

This study reveals a worrying increase in mortality from Alzheimer's disease (AD) and other dementias in Ecuador between 2012 and 2022, especially among women, adults over 65 years of age, and residents of the Sierra region. Several factors that could negatively influence cognitive function were observed. These findings are consistent with global trends and suggest that biological, environmental, and social variables, such as aging, postmenopausal hormonal changes, chronic exposure to hypoxic altitude conditions, and unequal access to health services, could play a key role in this disease.

**Conclusion:**

Mortality from Alzheimer's disease and other dementias in Ecuador showed a sustained increase between 2012 and 2022, reflecting a growing burden of these pathologies in the population and the urgent need to strengthen prevention, early diagnosis, and comprehensive treatment strategies. The disproportionate impact on women, adults over 65 years of age, and residents of the Sierra region suggests the involvement of various biological, environmental, and social determinants of health, which requires more rigorous surveillance and a differentiated approach for these particularly vulnerable populations.

## 1 INTRODUCTION

Alzheimer’s disease (AD) and other forms of dementia represent a growing burden in public healthcare worldwide, especially in the current context of accelerated and extended population aging around the globe and its repercussions on the prevalence of neurodegenerative diseases. This growth is linked not only to increased life expectancy due to medical advancements, but also to greater awareness and diagnosis of these pathologies. Dating back to 1906 and the first description by Dr. Alois Alzheimer, the understanding of these diseases has been evolving continually, although significant challenges remain in their management. Dementias, including AD, are progressive neurodegenerative diseases that compromise memory, thinking, and behavior, ultimately leading to the loss of functional capacity and death [1, 2].

Epidemiologically, AD is the most common cause of dementia, accounting for 60% to 80% of all cases. According to the World Health Organization (WHO), over 55 million people worldwide live with dementia, and this figure is expected to triple by 2050 due to predictions regarding population aging. In terms of prevalence, dementia affects approximately 5-8% of people over 60 years of age, and the incidence is equally concerning, with nearly 10 million new cases diagnosed each year [3].

In Latin America, and specifically in Ecuador, this situation has significantly shifted due to public health reforms implemented at the beginning of the 21st century. The preceding modifications have improved access to medical services and promoted health programs for older adults. However, despite these advances, disease-specific resources for the diagnosis and treatment of dementia have been insufficient. In Ecuador, the prevalence of dementia is estimated at approximately 8.5%, and the number of affected individuals is projected to grow exponentially in the coming decades due to demographic changes and increased life expectancy. In this context, the lack of well-founded, rigorous, and detailed studies on mortality in patients affected by these diseases is evident in the scientific community [1, 4].

Recently, several studies have highlighted the growing burden of dementia in middle- and low-income countries, where healthcare systems are often less prepared to handle such complex pathologies. Ecuador, alongside its geographic and socioeconomic diversity, offers an interesting context for exploring how these variables may influence mortality rates from various types of dementia [5].

This study aligns with the “Mental Health and Neuroscience” research subline at the Universidad Católica de Cuenca, providing a comprehensive framework for studying mental disorders from their pathophysiology to clinical evaluation. The focus is aimed at detailing the clinical and neuropsychological manifestations of dementia in Ecuador, as well as refining diagnostic and therapeutic tools. Therefore, the purpose of this study is to analyze mortality from Alzheimer's disease and other dementias in Ecuador during the period between 2012 and 2022, a vital approach to better understand the magnitude of the problem in this country, as well as identify possible risk factors and areas for improvement.

## 2 MATERIALS AND METHODS

A retrospective, descriptive time-series analysis was conducted on adult subjects diagnosed with Alzheimer’s disease (AD) and other forms of dementia in several geographical regions of Ecuador, covering the period from 2012 to 2022.

Ecuador is divided administratively into parishes, which form cantons, provinces, and administrative regions. The country was divided into four regions: Coast, Sierra, Eastern, and Insular, to simplify the geographic interpretation of results.

The definition of mortality due to AD and other dementias was based on the inclusion of these diagnoses as either the primary or a contributing cause of death in death certificates recorded by the National Institute of Statistics and Census (INEC). Mortality rates were calculated using the number of deaths registered in the standardized forms from INEC and census data from 2022, published by the same institution. The sample was selected using intentional non-probability sampling.

The inclusion criteria were as follows:

• Death records specifying AD or other dementias as the primary or contributing cause of death between 2012 and 2022.

• Complete records containing age, sex, province of residence, and date of death.

The exclusion criteria were as follows:

• Incomplete or ambiguous records that did not specify AD or related dementias.

• Cases lacking sufficient information to determine the cause of death.

• Deaths recorded abroad.

Subjects were evaluated according to age group, sex, and geographical region. The study adhered to ethical research principles, with approval from the university's ethics committee (CEISH), approval date: August 2^nd^, 2022, and protocol number: CEISH-UCACUE-2024-082. Data confidentiality was maintained, and no direct personal information from the individuals was collected.

### 2.1 Statistical Analysis

The results were expressed as absolute numbers and percentages for qualitative variables. Incidence rates were calculated as the sum of all new episodes of AD or other dementias divided by the size of the population every year. Data analysis was performed using IBM SPSS version 17.0 (SPSS Inc., Chicago, USA).

## 3 RESULTS

**Fig. (1):**
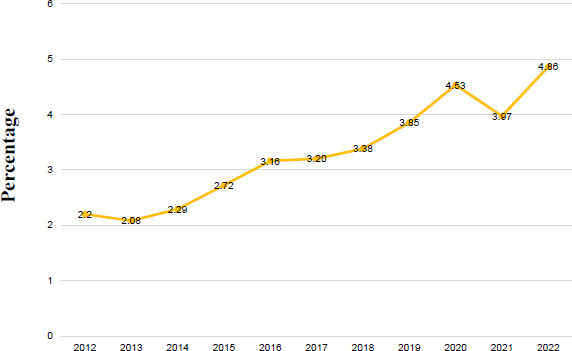
National mortality rate from Alzheimer's disease and other dementias (2012-2022).

The study included 855,122 subjects evaluated from 2012 to 2022, out of which 4,836 were identified as AD-related deaths (0.56%) and 1,317 as deaths due to other forms of dementia (0.15%). National mortality rates showed a solid upward trend over the decade, rising from 2.2 deaths per 100,000 inhabitants in 2012 to 4.86 per 100,000 inhabitants in 2022 (Fig. **1**). Among those who died from AD, 1,828 were men and 3,008 were women. Similarly, for other dementias, 551 were men and 766 were women. The annual distribution of deaths according to sex and age group is shown in Table **1** for AD and Table **2** for other dementias. Additionally, the annual regional distribution of deaths from AD and other dementias is provided in Tables **3** and **4**, respectively. Remarkably, the Sierra was the most affected region in both groups.

## 4 DISCUSSION

On a global scale, according to the 2019 Global Burden of Disease (GBD) study, the number of people living with dementia is expected to rise from 57.4 million in 2019 to 152.8 million by 2050. This staggering growth is mostly driven by population aging and demographic expansion. The previous behavior is paralleled by the rising mortality rates observed in countries like Mexico, where AD-related deaths have been steadily growing in the past five years. Similarly, in the United States, AD ranked as the sixth leading cause of death in 2021, while in China, the mortality rate from AD and other dementias has also shown an upward trend [6].

Our findings align with these global observations, not only indicating a robust increment in the mortality rate from AD and other dementias but also highlighting the disproportionate impact on women, individuals over 65 years of age, and residents of the Sierra region, especially in densely populated provinces, such as Pichincha and Guayas, where the most significant statistical differences were reported. This pattern suggests that these populations may require closer monitoring and more comprehensive evaluation to account for comorbidities and other potentially life-threatening conditions that could influence both survival rates and patients’ quality of life [7]. From a molecular perspective, cellular senescence is a state of permanent cell growth arrest and is believed to contribute importantly to several aging-related diseases, including AD [8]. Senescent astrocytes, microglia, endothelial cells, and neurons have been detected in the brains of AD patients and AD animal models. Due to the therapeutic potential of reducing the senescent cells burden to extend healthspan and delay the onset of age-related diseases, there is growing interest in developing senotherapeutic strategies that incorporate multidisciplinary technologies from diverse fields, such as biology, chemistry, nanotechnology, and immunology; this approach includes conventional senotherapeutics, prodrugs, protein degraders, nanocarriers, and immunotherapies [9, 10].

The higher mortality rates observed in women are consistent with trends reported in other countries, suggesting a global tendency for females to experience greater susceptibility to neurodegenerative diseases. Additionally, menopause has a negative effect on metabolism and memory and could reduce protection against cognitive impairment and dementia in terms of vascular health. These gender disparities in mortality rates also include higher rates of complications, such as diabetes, hypertension, and a greater risk of coronary heart disease [11, 12]. These factors should be analyzed in future studies in this population. In the United States, for instance, women showed a higher mortality rate from AD in 2019, with an age-adjusted rate of 263.0 per 100,000 for women aged 65 and older, compared to 186.3 per 100,000 for men [13]. Similarly, in China, the annual increase in AD-related mortality rates from 1990 onward was substantial for both men (2.70%) and women (2.29%) due to the heavy influence of rapid urbanization and population aging [14]. Reports from Germany and Japan reflect similar trends, where women not only have a higher prevalence of AD but also face higher mortality rates, likely due to longer life expectancy and greater biological vulnerability to the disease [15].

Additionally, our study found higher mortality rates in individuals residing in Ecuador’s Sierra region, which is characterized by an average altitude exceeding 3,000 meters above sea level. The increased prevalence in this region may be linked to environmental, biological, and socioeconomic factors that uniquely affect those living at such altitudes. A study by Aboouf *et al.* explored how high altitudes affect brain function due to hypoxia, namely reduced blood oxygen saturation (SpO_2_) levels and impaired memory and attention. SpO_2_ decreases as altitude rises, from 96% at 2,500 meters to 81% at 5,100 meters. At altitudes above 2,500 meters, lower oxygen pressure may impair cognition, mood, and neurocognitive performance [16]. Long-term exposure to a high-altitude hypoxic environment affects cognitive function, which is manifested in features, such as attention, memory ability, and inhibitory control. Another mechanism involves hyperplasia of red blood cells, which leads to cumulative changes in brain structure and local fine tissue structure and function, thus negatively affecting cognitive function and reducing cognitive level [17, 18].

In addition to geographical factors, health determinants, such as socioeconomic status and access to healthcare services, are likely to play a critical role in mortality rates. For example, studies from the United States show that AD-related mortality varies significantly by state. The lowest rates were observed in New York (13.1 deaths per 100,000 people), while the highest were reported in South Dakota and Mississippi (56 and 55.8 deaths per 100,000 people, respectively). These differences may reflect disparities in healthcare access and the development of care and prevention programs [8]. Similarly, a study conducted in Mexico by Gómez *et al.* reported mortality rates that varied across regions, ranging from 25.2 deaths per 100,000 inhabitants in Mexico City to 10.1 in Chiapas and 12.3 in Oaxaca, indicating a heavier burden of mortality in certain Mexican regions compared to Ecuador [19].

Therefore, it is necessary to promote strategies and early intervention policies for high-risk groups that include specialized consultations aimed at the early identification of cognitive impairment, with timely therapeutic management of the condition and its comorbidities, especially cardiovascular ones. Likewise, it is essential to promote the creation of support networks in primary healthcare to ensure the prevention of the main risk factors for dementia, most of which are modifiable from an early age [20].

Despite these insights, it is important to acknowledge the limitations of this study. The most significant setback of this report is its retrospective design, which could evoke potential biases and inaccuracies related to underreporting, especially in relation to the use of death certificate data, which would lead to the potential underestimation of this outcome variable. Moreover, the absence of a detailed breakdown between urban and rural zones makes detailed comparative analysis significantly more difficult. Finally, the lack of specific health determinants, such as socioeconomic status and healthcare accessibility, which could influence the epidemiological behavior of AD and other dementias, must be considered when interpreting the findings and planning future research.

## CONCLUSION

In conclusion, mortality from AD and other dementias in Ecuador showed a significant increase from 2012 to 2022. This continuous rise reflects the growing burden of these diseases in this country, highlighting the urgent need to improve strategies for the prevention, diagnosis, and treatment of such conditions. The predominant impact on women, individuals over 65 years of age, and those residing in the Sierra region underscores the importance of closely monitoring these populations, mainly focusing on comorbidities and the disease itself. Aging, the risk profile of women during menopause, and a high-altitude hypoxic environment may be key factors contributing to the higher mortality in these groups. Additionally, this pattern demonstrates the necessity of addressing both environmental and socioeconomic factors in future research, as these variables may influence the prevalence and mortality of AD and other dementias across different regions of Ecuador and in other similar global contexts.

## Figures and Tables

**Table 1:** Annual distribution of deaths from alzheimer's disease by sex and age group (2012-2022).

**Variables**	**2012**	**2013**	**2014**	**2015**	**2016**	**2017**	**2018**	**2019**	**2020**	**2021**	**2022**
**Men (n, %)**	109 (38.93%)	106 (38.55%)	34 (34.34%)	130 (33.94%)	168 (38.44%)	158 (35.35%)	164 (34.38%)	229 (38.68%)	261 (42.23%)	220 (39.22%)	249 (37.33%)
**Women (n, %)**	171 (61.07%)	169 (61.45%)	65 (65.66%)	253 (66.06%)	269 (61.56%)	289 (64.65%)	313 (65.62%)	363 (61.32%)	357 (57.77%)	341 (60.78%)	418 (62.67%)
**15-49 years (n, %)**	0 (0.00%)	1 (0.36%)	1 (1.01%)	0 (0.00%)	0 (0.00%)	0 (0.00%)	0 (0.00%)	1 (0.17%)	1 (0.16%)	1 (0.18%)	2 (0.30%)
**50-64 years (n, %)**	8 (2.86%)	6 (2.18%)	3 (3.03%)	9 (2.35%)	10 (2.29%)	2 (0.45%)	7 (1.47%)	4 (0.68%)	13 (2.10%)	13 (2.32%)	5 (0.75%)
**65 years and older (n, %)**	272 (97.14%)	268 (97.45%)	95 (95.96%)	374 (97.65%)	427 (97.71%)	445 (99.55%)	470 (98.53%)	587 (99.16%)	604 (97.73%)	547 (97.50%)	660 (98.95%)
**Total (n, %)**	280 (100%)	275 (100%)	99 (100%)	383 (100%)	437 (100%)	447 (100%)	477 (100%)	592 (100%)	618 (100%)	561 (100%)	667 (100%)

**Table 2:** Distribution of deaths from other dementias by sex and age group (2012-2022).

**Variables**	**2012**	**2013**	**2014**	**2015**	**2016**	**2017**	**2018**	**2019**	**2020**	**2021**	**2022**
**Men (n, %)**	23 (37.70%)	23 (43.40%)	110 (41.04%)	26 (43.33%)	44 (51.16%)	38 (42.22%)	42 (42.42%)	24 (32.88%)	84 (48.00%)	56 (38.89%)	81 (38.94%)
**Women (n, %)**	38 (62.30%)	30 (56.60%)	158 (58.96%)	34 (56.67%)	42 (48.84%)	52 (57.78%)	57 (57.58%)	49 (67.12%)	91 (52.00%)	88 (61.11%)	127 (61.06%)
**15-49 years (n, %)**	0 (0.00%)	0 (0.00%)	0 (0.00%)	0 (0.00%)	0 (0.00%)	1 (1.11%)	0 (0.00%)	0 (0.00%)	0 (0.00%)	0 (0.00%)	0 (0.00%)
**50-64 years (n, %)**	4 (6.56%)	2 (3.77%)	5 (1.87%)	1 (1.67%)	3 (3.49%)	0 (0.00%)	1 (1.01%)	1 (1.37%)	1 (0.57%)	3 (2.08%)	1 (0.48%)
**65 years and older (n, %)**	57 (93.44%)	51 (96.23%)	263 (98.13%)	59 (98.33%)	83 (96.51%)	89 (98.89%)	98 (98.99%)	72 (98.63%)	174 (99.43%)	141 (97.92%)	207 (99.52%)
**Total (n, %)**	61 (100%)	53 (100%)	268 (100%)	60 (100%)	86 (100%)	90 (100%)	99 (100%)	73 (100%)	175 (100%)	144 (100%)	208 (100%)

**Table 3:** Annual distribution of deaths from alzheimer's disease by regions in ecuador (2012-2022).

**Date**	**Coast (n, %)**	**Sierra (n, %)**	**Eastern (n, %)**	**Insular (n, %)**	**Total (n, %)**
**2012**	128 (6.69%)	147 (5.12%)	5 (10.42%)	0 (0.00%)	280 (100%)
**2013**	94 (4.92%)	180 (6.27%)	1 (2.08%)	0 (0.00%)	275 (100%)
**2014**	35 (1.83%)	61 (2.13%)	3 (6.25%)	0 (0.00%)	99 (100%)
**2015**	158 (8.26%)	221 (7.70%)	3 (6.25%)	1 (16.67%)	383 (100%)
**2016**	159 (8.32%)	277 (9.65%)	1 (2.08%)	0 (0.00%)	437 (100%)
**2017**	172 (9.00%)	275 (9.58%)	0 (0.00%)	0 (0.00%)	447 (100%)
**2018**	172 (9.00%)	302 (10.52%)	3 (6.25%)	0 (0.00%)	477 (100%)
**2019**	219 (11.45%)	364 (12.68%)	6 (12.50%)	3 (50.00%)	592 (100%)
**2020**	276 (14.44%)	335 (11.67%)	7 (14.58%)	0 (0.00%)	618 (100%)
**2021**	221 (11.56%)	330 (11.50%)	9 (18.75%)	1 (16.67%)	561 (100%)
**2022**	278 (14.54%)	378 (13.17%)	10 (20.83%)	1 (16.67%)	667 (100%)
**Total**	**1912 (100%)**	**2870 (100%)**	**48 (100%)**	**6 (100%)**	**4,836 (100%)**

**Table 4:** Annual distribution of deaths from other dementias by regions in ecuador (2012-2022).

**Date**	**Coast (n, %)**	**Sierra (n, %)**	**Eastern (n, %)**	**Insular (n, %)**	**Total (n, %)**
**2012**	6 (1.60%)	54 (5.83%)	1 (5.88%)	0 (0.00%)	61 (100%)
**2013**	13 (3.48%)	40 (4.32%)	0 (0.00%)	0 (0.00%)	53 (100%)
**2014**	103 (27.54%)	164 (17.71%)	1 (5.88%)	0 (0.00%)	268 (100%)
**2015**	27 (7.49%)	32 (3.46%)	0 (0.00%)	0 (0.00%)	60 (100%)
**2016**	42 (11.23%)	44 (4.75%)	2 (11.76%)	0 (0.00%)	86 (100%)
**2017**	33 (8.92%)	64 (6.91%)	2 (11.76%)	0 (0.00%)	90 (100%)
**2018**	36 (9.55%)	44 (4.43%)	1 (5.88%)	0 (0.00%)	99 (100%)
**2019**	8 (2.14%)	64 (6.91%)	1 (5.88%)	0 (0.00%)	73 (100%)
**2020**	36 (9.63%)	134 (14.47%)	5 (29.41%)	0 (0.00%)	175 (100%)
**2021**	26 (6.93%)	117 (12.63%)	2 (11.76%)	0 (0.00%)	144 (100%)
**2022**	34 (9.09%)	171 (18.47%)	2 (11.76%)	0 (0.00%)	208 (100%)
**Total**	**374 (100%)**	**926 (100%)**	**17 (100%)**	**0 (0.00%)**	**1317 (100%)**

## Data Availability

The data sets used and/or analysed during this study are available from the corresponding author [C.E] upon request.

## LIST OF ABBREVIATIONS

AD: = Alzheimer's Disease
INEC: = National Institute of Statistics and Censuses
WHO: = World Health Organization
GBD: = Global Burden Disease
